# Hemoglobinopathy prevention in primary care: a reflection of underdetection and difficulties with accessibility of medical care, a quantitative study

**DOI:** 10.1038/s41431-022-01051-8

**Published:** 2022-02-25

**Authors:** Margo E. van Vliet, Jean-Louis H. Kerkhoffs, Cornelis L. Harteveld, Elisa J. F. Houwink

**Affiliations:** 1 (Bio-)Medical Faculty, Leiden University Medical Center, Postal Zone V0-P, PO Box 9600, 2300RC Leiden, The Netherlands; 2 Department of Hematology, HAGA Hospital, Els Borst Eilersplein 275, 2545AA The Hague, The Netherlands; 3 Laboratory for Diagnostic Genome Analysis, Department of Clinical Genetics, Leiden University Medical Center, Postal Zone S6-P, PO Box 9600, 2300RC Leiden, The Netherlands; 4 Department of Public Health and Primary Care, Leiden University Medical Center, Postal Zone V0-P, PO Box 9600, 2300RC Leiden, The Netherlands

**Keywords:** Population screening, Anaemia

## Abstract

As in most Northern European countries, the prevalence of hemoglobinopathies in The Netherlands is increasing due to migration. Although hemoglobinopathies are severe chronic diseases with few treatment options, timely detection of carriers allows at-risk couples to make informed reproductive choices such as pre-implantation diagnosis, prenatal diagnosis or termination of affected pregnancies. Using a quantitative design, we evaluated the prevalence of hemoglobinopathies in The Hague region, The Netherlands. Patient and carrier registries from hospital, laboratory and general practitioners allowed this quantitative analysis. The highest prevalence of hemoglobinopathies was seen in immigrant neighborhoods, and a large gap was noted between estimated carrier prevalence and the actual registration of carriers in electronic patient records. Carrier prevalence was estimated to be 13,704; however, the ELAN database contains only 1542 cases with ICPC codes for sickle cell disease or thalassemia. Although more research is needed to define the requirements of the healthcare system to address this challenge, this study clearly shows the gap between estimated carrier prevalence and registration and thereby the pressing need for action.

## Introduction

Hemoglobinopathies (HBPs) are the most common of all monogenic autosomal-recessive diseases and the most severe include sickle cell disease and alpha/beta-thalassemia major. As carriers are typically asymptomatic and unaware of their carrier status, carrier couples often do not realize that with each pregnancy they face a 25% risk of a child with HBP.

HBP is caused by genetic defects in the globin genes that encode the hemoglobin protein. Whereas thalassemia patients show defects in globin gene expression, sickle cell patients express globin proteins with an altered structure. In both cases, the genetic defects have severe consequences for patients [1]. Sickle cell disease results in recurrent vaso-occlusive crisis, which can cause acute and chronic complications that may result in disability. The treatment for sickle cell disease consists of prophylactic antibiotics, folic acid, analgesics and in severe cases exchange blood transfusions and hydroxyurea. Beta-thalassemia results in severe anemia, which is treated with blood transfusions and iron chelation therapy. The only curative treatment for sickle cell disease and beta-thalassemia is hematopoietic stem cell transplantation. Unfortunately, finding a compatible stem cell donor is complex and may be complicated by graft-versus-host disease [2]. Gene therapy is still experimental, expensive and not accessible for the majority of patients.

HBPs are traditionally endemic in countries where malaria, especially *Plasmodium falciparum* malaria, is or was common, as heterozygous carrier status has a protective effect on death from falciparum malaria [3]. As migration increases worldwide, HBP is now more common in countries without a history of malaria, such as the Netherlands, and around nine HBP gene homo- and compound heterozygotes are now born for every 10,000 migrant births in the Netherlands [4]. Compared to other countries in the European Union, in 2006 the Netherlands also had the highest proportion of residents and pregnant women in groups at high risk for HBP [5], and following increased migration since 2006 the number of people at risk is likely to be higher today [6].

Screening for carriers of HBP requires action at several stages of gene transmission and while primary screening for HBP or HBP traits has yet to be implemented in the Netherlands, secondary screening by the heel prick blood spot, a blood sample taken in the first week after birth, has been in place since 2007. A weakness of this test is that it mainly detects homozygous and heterozygous carriers of sickle cell allele (HbS) rather than beta-thalassemia carriers, as the percentage of hemoglobin A2 becomes detectably increased after the first 6 months after birth. Around 800 HbS heterozygotes and 35–40 babies are detected with sickle cell disease or beta-thalassemia per year (in 2018, 11 beta-thalassemia intermedia/major and 31 sickle cell disease) [7]. Carrier screening prior to conception (preconception screening) or early antenatal screening are alternatives to neonatal screening. It provides options for prenatal- and antenatal diagnostics and allows carrier couples to make informed reproductive choices, reducing the risk of being confronted with a child with HBP unexpectedly [8].

Several countries in Europe have implemented early antenatal screening programs for HBP and a good example is the United Kingdom, which has implemented a national prevention program that tests all pregnant women in high prevalence areas and performs a blood cell count in all pregnant women living in those high prevalence areas in early pregnancy. In low prevalence areas, the family ancestry is questioned and the women with a high risk on HBP are detected [9]. In some Mediterranean countries prevention programs have achieved almost 90% declines in incidence [10]. In some regions, such as Southern Italy and Southern France, prevention programs begin at secondary school ages [11].

The aim of this study was to explore better strategies for HBP prevention in the preconception and antenatal phase in general practice, using both quantitative and qualitative analysis. This article focusses on the quantitative part. Our aim was to identify the number of carriers and affected births for sickle cell disease and beta-thalassemia already registered and estimate the expected births from the number of births in the ethnic minority populations and compare these figures to the numbers actually registered in the city of the Hague and surrounding areas. This will reflect the awareness and competences of Dutch physicians in primary care and the need for population health management (PHM). Population health is defined as the health outcomes of a group of individuals and the distribution of these outcomes within the group. In the Netherlands, the general practitioner (GP) plays an important gatekeeper role at this phase and through shared decision making together with the couple decides whether a referral or extra tests are necessary. The GP therefore has the first opportunity to detect a carrier of HBP in collaboration with other primary care specialists. We explored the gap between registered and expected HBP carriers by first quantifying the prevalence of HBP and HBP trait in the multi-ethnic city of The Hague and its surroundings.

## Methods

We carried out a prospective quantitative study based on data from the Hemoglobinopathies Reference Lab of the Leiden Diagnostic Genome Analysis laboratory (LDGA) at Leiden University Medical Center (LUMC), the Extramural LUMC Academic Network (ELAN) and the HBP patient database at the HAGA hospital in The Hague.

The quantitative aspect of the study was carried out in The Hague and its surroundings in July 2020. Statistical analysis was performed using the Statistical Package for the Social Sciences (SPSS), version 26.0. To calculate prevalence, we used the HAGA database containing all patients with beta-thalassemia intermedia/major and sickle cell disease in The Hague region. Carrier prevalence was calculated using the information provided by the Hemoglobinopathies Reference Lab within the LDGA at the Clinical Genetics department of the LUMC and the ELAN database. LUMC/HAGA is an acknowledged Expert Centre for Hemoglobinopathy and the HBP Reference Lab is part of the LDGA. This lab provides HBP diagnostic services to the whole country. The ELAN database contains data from GPs in the region of Leiden and The Hague. The ELAN database is linked to GPs in the region of the LUMC. The database contains all International Classification of Primary Care (ICPC) codes the GPs link to a note in a patient dossier; these ICPC codes are thus no official diagnosis. The ICPC codes can also be used if a GP suspects the diagnosis. Patients were grouped by neighborhood, allowing the analysis of prevalence and linking to ethnic diversity in those neighborhoods. To assess possible missing patients and carriers and estimate carrier prevalence, we compared the number of registered patients in the GP database for The Hague region (ELAN database) with the number in the HAGA hospital database.

## Results

**Fig. 1:**
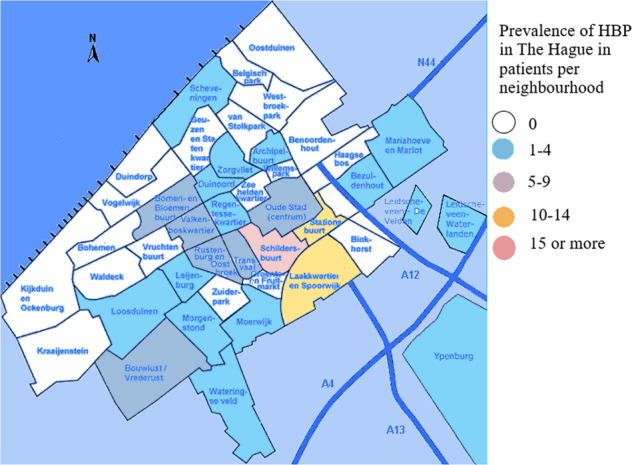
Prevalence of HBP patients in The Hague.

**Fig. 2:**
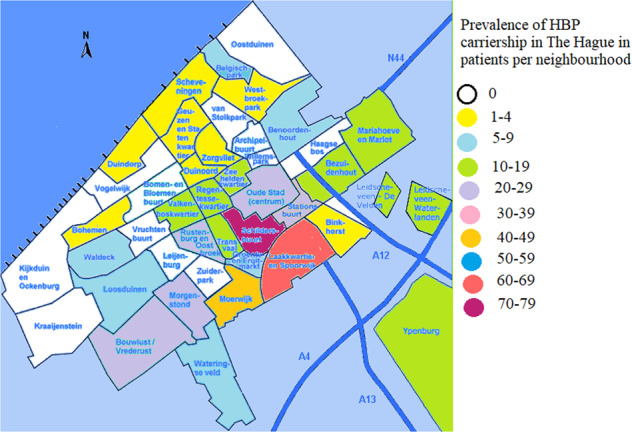
Prevalence of HBP carriership in The Hague.

**Fig. 3:**
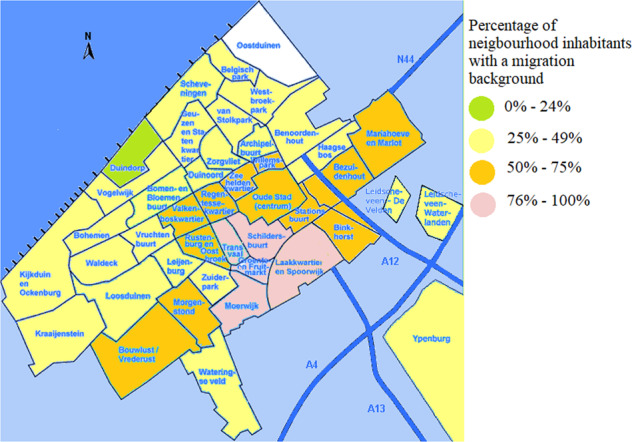
Inhabitants with a migration background in The Hague.

A total of 162 patients with sickle cell and beta-thalassemia intermedia/major were treated in the HAGA hospital, The Hague in June 2020, of whom 67.9% (*n* = 113) had a GP in the city of The Hague. The remaining 49 patients mostly had GPs in nearby cities such as Zoetermeer (*n* = 9), Rijswijk (*n* = 6) and Delft (*n* = 5). In one case the GP location was unknown. In The Hague, more than half (57.3%) of patients mentioned a GP in the city districts “Centrum” or “Escamp”, and at the level of city neighborhoods the highest prevalences of HBP were noted for the “Schildersbuurt” (*n* = 16), the “Laakkwartier and Spoorwijk” (*n* = 11) and the “Stationsbuurt” (*n* = 10). The distribution of HBP patients can be seen on a map of The Hague in Fig. 1. Figure 2 visualizes the percentage of HBP carriers per neighborhood, based on the postal codes of patients found in the LDGA database. Between 2006 and 2020, 3664 HBP carriers have been recorded in the LDGA database, of whom 470 had a Hague city postal code, with the highest prevalence seen in the “Schildersbuurt” (*n* = 71), the “Laakkwartier and Spoorwijk” (*n* = 62) and “Moerwijk” (*n* = 46). Figure 3 illustrates the distribution of inhabitants with a migration background (western and non-western) in The Hague (54,7%, *n* = 297,987), which correlates with high levels of carriers and patients with HBP. The correlation coefficient *R*^2^ is 0.5, which is moderate.

In July 2020, “ELAN”, the primary care physician information system (HIS or electronic patient record) database for all physicians affiliated to the LUMC, included data on 556,835 patients. Of these patients, 0.35% (*n* = 1969) received the ICPC code B78 for hereditary hemolytic disease, divided into 1191 codes for thalassemia (B78.01), 351 for sickle cell anemia (B78.02), 35 for G6PD deficiency (B78.03) and 392 not further defined. People with a B78 code mainly originating from The Hague (41.4%), Zoetermeer (38.9%), Leiden (5.8%) Alphen aan den Rijn (4.4%) and other places surrounding The Hague (9.5%). An important side note is the fact that ICPC codes are used for carriers, patients and even for suspected carriers or patients.

In July 2020, the LDGA database contained 4397 registrations of beta-thalassemia or sickle cell mutations confirmed by DNA analysis, with registrations tracked since 2006. This included 662 sickle cell disease patients and 71 beta-thalassemia intermedia/major patients, and a total of 3664 carriers of disease alleles (1808 SCD; 1856 beta-thalassemia intermedia/major). Patients were living in The Netherlands, The Netherlands Antilles, Belgium and Germany of whom 62 patients were residents of The Hague at the time of diagnosis and 104 patients were located in the other residential areas mentioned above.

In 2018, 11 positive cases of beta-thalassemia intermedia/major and 31 cases of sickle cell disease were found thanks to heel prick screening in The Netherlands; this in the context of screening that included 54,610 newborns with a mother with a migration background [12]. An estimation is that 10% is from a high-risk ancestry, thus 5461 births with a high risk of clinically severe forms of HBP. Using the Hardy–Weinberg equilibrium, we calculate that 873 babies born in 2018 carried a sickle cell or beta-thalassemia allele. Since the number of births registered from the heel prick screening is stable at around 35–40 births a year since 2007, we expect around 800–900 babies with HBP trait a year. If we calculate back to 2000, there would have been born around 14,400 and 16,200 carriers of sickle cell disease or beta-thalassemia in the past 18 years. As 6334 babies were born in The Hague in 2019, of which 3619 had at least one parent with a migration background, we estimate that 58 HBP carriers were born The Hague in 2019 [13]. Around 800 carriers of sickle cell disease alleles are reported each year following heel prick screening, which is in accordance with the calculations we performed [14]. In proportion to migration-related births, we estimate that around 53 children with HbS trait are born each year in The Hague.

Combining data from the ELAN, HAGA and LDGA databases, we then calculated the estimated carrier prevalence in The Netherlands in people from a migration background based on the Hardy–Weinberg Equilibrium. Table 1 shows figures for variables and the calculated carrier prevalence, including the striking carrier prevalence figure based on the HAGA database: 11,328 HBP carriers in the city of The Hague. All patient and carrier prevalences are stated in overview Table 2.

**Table 1:** Hardy–Weinberg calculations (p2 + 2pq + q2 = 1)^a^ for the prevalence of HBP carriership.

*x*	q^b^	p^c^	2pq	Carrier prevalence
The Hague by LDGA database	0.0145	0.9855	0.02859	8426
The Hague by HAGA database	0.0196	0.9804	0.0384	11,328
Main residential places ELAN database by LDGA database	0.01495	0.9851	0.02945	13,704

^a^p^2^ is the frequency of the homozygote dominant genotype, 2pq is the frequency of the heterozygous genotype, q^2^ is the frequency of the homozygous recessive genotype.

^b^√Total patients/total inhabitants with migration background. The Hague: LDGA database 62 patients, HAGA database 113 patients, 294,744 inhabitants with migration background. Residential places ELAN database: 104 patients, 465,267 inhabitants with migration background.

^c^1–q.

**Table 2:** Overview of patient and carrier prevalence.

0	Homozygous beta-thalassemia and sickle cell disease patients	Carrier prevalence beta-thalassemia and sickle cell disaese calculated by Hardy–Weinberg
The Hague by LDGA database	62^a^	8426
The Hague by HAGA database	113^a^	11,328
Main residential places ELAN database	Unknown	13,704
The Hague by Neonatal screening 2018	Unknown	58 births per year
The Netherlands by Neonatal screening 2018	42^a^	873 births per year

^a^Absolute numbers.

## Discussion

In this study, we evaluated the size of the problem about HBP care in primary care. We performed database research to visualize HBP prevalence in The Hague and calculated carrier prevalence. We found that HBP patients and HBP carriers were both mainly concentrated in relatively few neighborhoods in The Hague, which can be explained by the high percentages of inhabitants with a migration background in those neighborhoods.

Hardy–Weinberg calculations gave an estimated prevalence of HBP trait of 8426 based on the LDGA database and 11,328 based on the HAGA database. The difference probably relates to the fact that the LDGA database only includes patients diagnosed from 2006 to 2020, whereas the HAGA database includes all living patients treated in the HAGA hospital to date of this study. Presumably, the prevalence estimate based on the LDGA database underestimates actual prevalence. As not all suspected carriers are sent to the Hemoglobinopathies Reference Lab of the LDGA. Part of the analysis is done by local peripheral clinical chemistry labs that do not consult the Hemoglobinopathies Reference Lab. As these labs report directly to the GP, it is expected that registration in the ELAN database takes place.

Many carriers are unknown to the GP and are often even unaware that they are carriers, an example of which is the small proportion (*n* = 470) of carriers from The Hague registered in the LDGA database, representing perhaps less than 5% of all carriers in the catchment area. This is important because undetected carriers face the risk of having a child with a clinically severe form of HBP. Many carriers escape detection which is further illustrated in the residential areas included in the ELAN database. Although carrier prevalence is estimated to be 13,704, the ELAN database contains only 1542 cases with ICPC codes for sickle cell disease or thalassemia. These numbers show that many carriers remain undiagnosed.

**Fig. 4:**
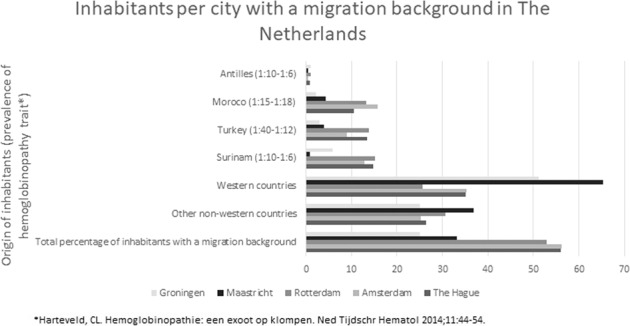
Inhabitants with a migration background in five big cities in The Netherlands.

Based on national heel prick data, an estimated 58 carriers were born in The Hague in 2019. Since the birth rate in The Hague has been stable in recent years, it is reasonable to estimate that around 60 carriers are born in The Hague every year. Therefore, since 2006 around 780 new carriers have been born, whereas only a total of 470 is registered in the LDGA database. The numbers for The Hague can be extrapolated to other cities in the Netherlands, such as Rotterdam and Amsterdam, since those cities have a similar percentage of inhabitants with a migration background (Rotterdam 52.3%; Amsterdam 55.6%; The Hague 54.7%). The inhabitants with a migration background are in the Hague mainly originating from Surinam, Morocco, Turkey and The Antilles. These origins are comparable with Amsterdam and Rotterdam, which is shown in Fig. 4.

However, the heel prick test is only performed by babies born in official health facilities. In the Netherlands there are about 40,000 undocumented inhabitants, for example, refused asylum seekers or illegal migrant workers. Necessary medical care is provided obligatory to this group because of European and Dutch conventions [15]. Nevertheless, the study of Hintjens et al. found that in The Hague and Rotterdam undocumented people are only seeking care in emergencies. Moreover, the undocumented population reported a fear of being detected, detained or even deported when they seek care whereas all Dutch medical facilities have a duty of confidentiality [16]. Therefore the number of births in inhabitants with a migration background can be underestimated.

### Future implications

In 1998, a screening program for HBP carriers was felt to be inappropriate in The Netherlands, based on a number of arguments including the low number of diagnosed children, a poor understanding of HBP in the at-risk population and difficulties concerning the best moment for screening [17]. A lot has changed since 1998, as the number of inhabitants with a migration background has increased by over 1 million and now (2020) accounts for 24.4% of the total population of The Netherlands [18]. In 2006, the World Health Organization published two resolutions on HBP, including recommendations for HBP screening and counseling programs [19], and the WHO now advises a preventive screening program when there are more than 20 affected births per year. As 42 affected births were detected by heel prick screening in The Netherlands in 2018, a screening program is thus recommended. However, the heel prick screening program alone is insufficient in the case of HBP disease because detection occurs after birth and the sequelae cannot be averted. An additional screening program such as preconceptional or antenatal screening to detect carriers is therefore needed.

The findings of this study can be extrapolated internationally. The underestimation of the carrier prevalence is already mentioned in 2010 by K.L. Hassell from the USA. She found a lack of registration of sickle cell patients and a paucity of data of sufficient quality. This study recommended standardized collection and centralized reporting to assess the real size of the problem, which is congruent with our findings [20].

To conclude, improving the health of populations from high-risk countries through PHM requires urgent primary prevention of HBP, such as preconception or antenatal screening. The problem is nevertheless still underreported by GPs and registration is not optimally used. However, the underdiagnosis of carriers can only be improved when there is a strong collaboration in all facets of primary care, for example, the midwives, children healthcare centers and genetic counselors. More research is needed to find out what difficulties the GPs and other primary care workers are facing and why the awareness is this low in The Netherlands. Currently, we are working on submitting results to EJHG of a qualitative study to explore the perspectives of GPs, carriers and patients on the low awareness in primary care in The Netherlands. Even though more research is needed to define the exact requirements of the Dutch healthcare system, this study clearly illustrates the size of the problem and the pressing need for action to increase informed reproductive decision making among a large at-risk population.

## Data Availability

The datasets generated and analyzed during the current study are available from the corresponding author on reasonable request.
